# Physical Properties of the Extracellular Matrix of Decellularized Porcine Liver

**DOI:** 10.3390/gels4020039

**Published:** 2018-05-01

**Authors:** Hiroyuki Ijima, Shintaro Nakamura, Ronald Bual, Nana Shirakigawa, Shuichi Tanoue

**Affiliations:** 1 Department of Chemical Engineering, Faculty of Engineering, Graduate School, Kyushu University, Fukuoka 819-0395, Japan; s.nakamura@kyudai.jp (S.N.); ronald.bual@g.msuiit.edu.ph (R.B.); shirakigawa@chem-eng.kyushu-u.ac.jp (N.S.); 2 Frontier Fiber Science and Technology, Faculty of Engineering, University of Fukui, Fukui 910-8507, Japan; tanoue@matse.u-fukui.ac.jp

**Keywords:** liver-specific extracellular matrix, gel, scaffold, tissue engineering, decellularization, solubilized extracellular matrix, physical property, storage modulus, loss modulus, biodegradation

## Abstract

The decellularization of organs has attracted attention as a new functional methodology for regenerative medicine based on tissue engineering. In previous work we developed an L-ECM (Extracellular Matrix) as a substrate-solubilized decellularized liver and demonstrated its effectiveness as a substrate for culturing and transplantation. Importantly, the physical properties of the substrate constitute important factors that control cell behavior. In this study, we aimed to quantify the physical properties of L-ECM and L-ECM gels. L-ECM was prepared as a liver-specific matrix substrate from solubilized decellularized porcine liver. In comparison to type I collagen, L-ECM yielded a lower elasticity and exhibited an abrupt decrease in its elastic modulus at 37 °C. Its elastic modulus increased at increased temperatures, and the storage elastic modulus value never fell below the loss modulus value. An increase in the gel concentration of L-ECM resulted in a decrease in the biodegradation rate and in an increase in mechanical strength. The reported properties of L-ECM gel (10 mg/mL) were equivalent to those of collagen gel (3 mg/mL), which is commonly used in regenerative medicine and gel cultures. Based on reported findings, the physical properties of the novel functional substrate for culturing and regenerative medicine L-ECM were quantified.

## 1. Introduction

Tissue engineering is a promising approach in realizing regenerative medicine, manifested by the combined use of cells, scaffolds, and growth factors. In particular, the reconstruction of the liver, which is the center of metabolism in our body, is one of the most important and difficult tasks in regenerative medicine. Herein, the scaffold is considered an exogenous pericellular factor that affects cells. Various materials have been developed as scaffolding materials for tissue engineering, and these have been broadly classified into natural and synthetic polymers. Natural biological macromolecules, such as collagen and laminin, are characterized by excellent cell signal transmission. For example, they are effective materials for the adhesion and proliferation of cells as well as for the expression of differentiation and functions.

In recent years, attention has been paid to decellularized tissues from which cellular components have been completely removed [1,2,3,4]. Since they are biologically derived materials, they possess excellent biocompatibility and capacity in maintaining the vascular network of tissues after decellularization. Therefore, they have been extensively studied as an organ template for transplantation. The recellularization of the decellularized tissue should be optimized in order to fabricate a functional reconstructed liver. Previous reports suggested that recellularized liver inoculated with hepatocytes expresses liver-specific functions such as albumin and urea synthesis [5,6]. Also, site-specific cell inoculation has been successfully performed [7]. However, the hepatocytes density of recellularized liver evaluated from the inoculum cell number was 10^6^–10^7^ cell/mL. These values are significantly below the level of 1–2 × 10^8^ cells/mL, which is considered to be the cell density in a healthy liver. In this regard, the formation of new liver tissue accompanied by cell growth within the organ template is essential. Also, recellularized liver transplantation experiments have not been successful for even a few days of cell engraftment [3,8]. Furthermore, in in vivo, cells are embedded in the ECM. Therefore, when the decellularized tissue is used as a scaffold material, it is difficult to reproduce a similar in vivo environment to that which existed before decellularization, where individual cells will be embedded in a similar manner within the ECM. In order to overcome the above problems, liver-specific ECM gel construction is indispensable as a suitable environment in the formation of functional liver tissue accompanied by angiogenesis [9,10]. Meanwhile, functional liver tissue formation accompanied with angiogenesis and hepatocyte proliferation has been reported in vivo by implanting cells with ECM model gel [7,10].

Recently, the maintenance and promotion of the liver phenotype [11,12,13,14] was reported to be effective when decellularized liver was solubilized (L-ECM) in the effort to construct a new culture substrate and generate a 3D injectable hydrogel platform for liver tissue engineering [15]. In addition, it is reported that the composition and structure of extracellular matrix (ECM) constituting each organization differs in accordance with the organ type [16,17]. Likewise, it is suggested that organ-specific ECM constitutes an optimal microenvironment around cells. Thus, the solubilized liver-specific ECM (L-ECM) is an effective substrate for different applications, from basic use in drug screening with hepatocyte culture to constructive application in liver regenerative medicine.

The behavior of cells is influenced by the components and structure of the scaffold, and by the material’s physical properties. For example, when hepatocytes were embedded using alginate gel with different elastic moduli (1 kPa, 12 kPa), increased albumin synthesis activity was elicited in conditions where the elastic modulus was 1 kPa. In addition, it was reported in the same study that increased albumin synthesis activity was elicited in conditions where the softest PEG-heparin material was used [18]. In other words, material properties are considered to be factors that affect cell behavior. However, cell adhesion, morphology, tissue formation ability, and functional expression are determined by the overall influence of the composition, surface characteristics, and mechanical properties of the substrate. Though there is a report on the mechanical properties of rat L-ECM [15], it is essential to consider the L-ECM of other animals (e.g., porcine) clinically and to examine their physical properties in detail.

For these reasons, it is important to quantify the physical properties of L-ECM. However, detailed investigations on them have not been reported. Therefore, in this study, L-ECM obtained by the solubilization of decellularized porcine liver, as well as L-ECM gel obtained by the spontaneous association of L-ECM, were prepared and their physical properties were evaluated.

## 2. Results and Discussion

### 2.1. Decellularization of Porcine Liver Tissue and Preparation of L-ECM

**Figure 1:**
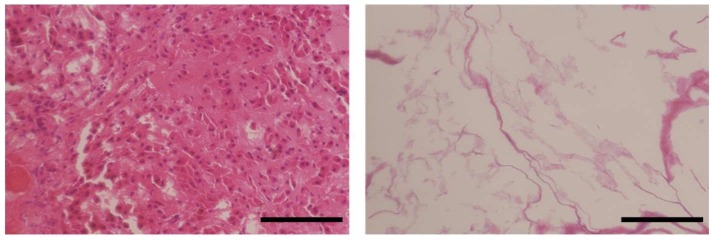
Histological observation (Hematoxylin and eosin staining) of native liver (**A**) and decellularized liver (**B**) (scale bars = 100 μm).

**Figure 2:**
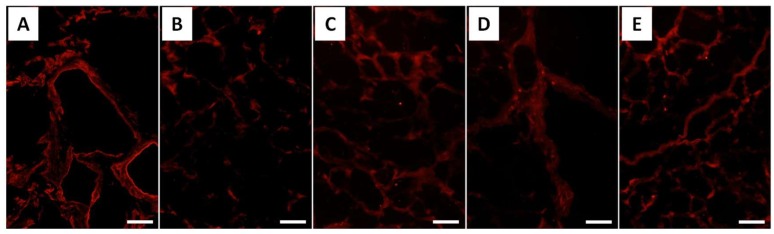
Immunolabeling of collagen types (**A**) I, (**B**) III, (**C**) IV, (**D**) V, and laminin (**E**), in decellularized samples with Triton solution. All images are displayed at a magnification of 20 times (scale bars = 50 µm).

**Figure 3:**
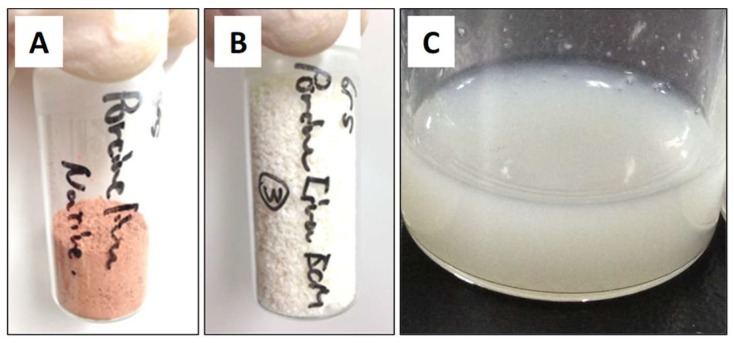
(**A**) Appearance of natural liver powder, (**B**) decellularized liver powder, and (**C**) L-ECM. Each powder type was obtained by the lyophilization of the corresponding liver tissue.

The immunohistological evaluation of porcine liver slices decellularized by Triton X-100 treatment was performed. In the histological evaluation of the liver after decellularization, the absence of the cellular cytoplasms and nuclei of cellular components was confirmed (Figure 1). Furthermore, since 92.4% of the DNA in the porcine tissue was removed in this treatment, it was confirmed that the decellularization of porcine liver slices by 1% Triton X-100 treatment was successful. Using immunohistological evaluations of this decellularized liver slice, collagen types I, III, IV, and V, and laminin originally contained in the native liver were detected (Figure 2). Despite the qualitative nature of fluorescence imaging, distinct red fluorescence was observed by staining using type I collagen antibodies. In addition, white powder was obtained by the lyophilization of this decellularized liver tissue. These series of processes lasted approximately 15 days and led to the harvesting of 18.0 ± 0.2 mg of decellularized porcine liver dry powder per 1 g (wet weight) of porcine liver tissue. Furthermore, by treating this powder with pepsin, L-ECM was obtained as liver-specific solubilized ECM (Figure 3). This L-ECM contained approximately 0.15 μg/mg of glycosaminoglycan (GAG), which was equivalent to the concentration yield of our previous study [11]. These results indicate that the acquisition of L-ECM as a solubilized substrate had a liver-specific matrix composition since all of the cellular components were removed.

### 2.2. Characterization of L-ECM

**Figure 4:**
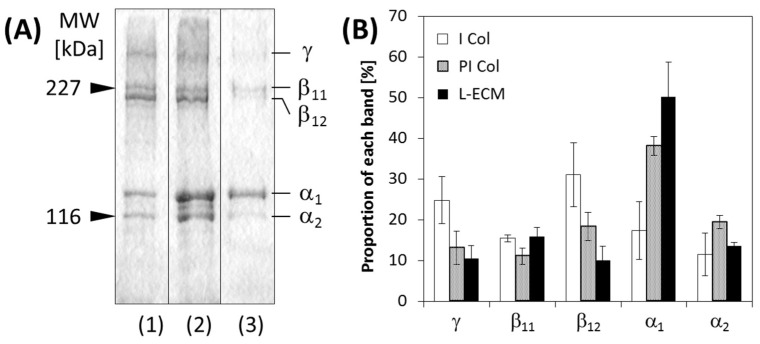
I (**A**) SDS-PAGE of each sample. (1) Solubilization with acid (I Col), (2) pepsin-acid solubilization (PI Col), and (3) L-ECM. The concentration of each solution was 0.75 mg/mL. (**B**) Ratios of detected bands in the all components (*n* = 3, bars represent standard deviation).

SDS-PAGE results and the signal ratios for each molecular weight band of all studied solutions are shown in Figure 4. Molecular weight bands were observed at MW = 116 and 227 kDa at all studied conditions (Figure 4A). Additionally, in each solution, differences in the relative densities of each molecular weight band were confirmed. Therefore, as shown in Figure 4B, the proportions of each molecular weight band for all studied molecules were quantified by image analyses using Image J. In the acid-solubilized porcine type I collagen (I Col) (Nitta Gelatin, Osaka, Japan), the γ and the β chains were the most prominent, while the proportion of the α chains was relatively small. Conversely, in pepsin-solubilized porcine type I collagen (PI Col) (Nitta Gelatin), the proportion of the α chains was large and the ratio of the γ and β chains was small. Additionally, the same trend was observed for L-ECM as that for the PI Col. In other words, it can be said that the L-ECM obtained in this study is a pepsin-solubilized liver-specific matrix substrate.

In the solubilization of collagen by pepsin, telopeptide is cleaved by hydrolysis [19]. Therefore, the γ chains, which is a trimer of the collagen chain and the dimerized β chains, are present in increased quantities in the acid-solubilized collagen. In contrast, the α chain, which is a single strand, exists in pepsin-solubilized collagen in increased quantities. Since L-ECM was pepsin-solubilized, the proportion of the α chains was the most prominent. Therefore, it was expected that pepsin removed telopeptides from the L-ECM. In type I collagen, the triple helix-forming site is composed of glycine–X–Y (X and Y represent other amino acids; proline and hydroxyproline are used in many cases). Conversely, since the telopeptide does not contain this type of repeating sequence and is a species-specific sequence for the animal that is being studied, it becomes the main antigenic site of collagen [19]. Therefore, the removal of telopeptides leads to the suppression of immune reactions so that pepsin-treated L-ECM is expected to become a biocompatible material for realizing tissue engineering technologies.

### 2.3. Rheological Properties of L-ECM Based on Dynamic Viscoelastic Evaluation

All of the rheological measurements were performed in wet conditions.

#### 2.3.1. Strain Dispersion Test

**Figure 5:**
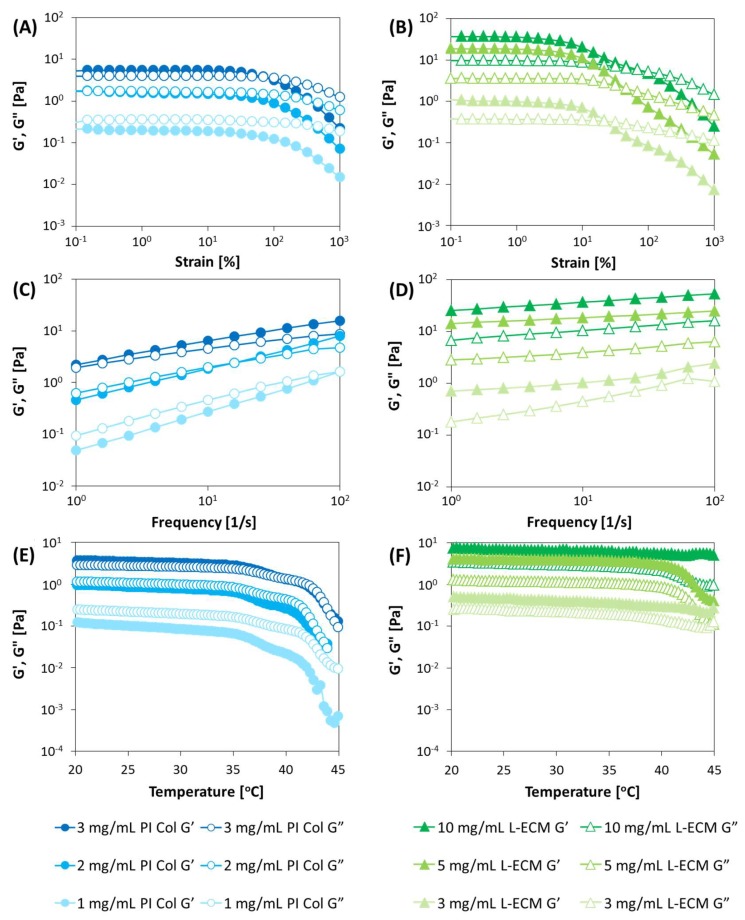
(**A**,**C**,**E**) Variations in G′ and G′′ at different concentrations of PI Col (3, 2, and 1 mg/mL), (**B**,**D**,**F**) L-ECM concentrations (10, 5, and 3 mg/mL). Corresponding variations of G′ and G′′ with (**A**,**B**) dynamic strain sweep (DSS) mode, (**C**,**D**) dynamic frequency sweep (DFS) mode, and (**E**,**F**) dynamic temperature ramp (DTR) mode measured by a rheometer (conditions for DSS: frequency 1 Hz, temperature 10 °C; conditions for DFS: strain 5%, temperature 10 °C; and conditions for DTR: frequency 1 Hz, and strain 5%). All samples were dissolved in an aqueous solution adjusted with HCl at pH = 3.0.

In the strain dispersion test (Figure 5A,B), PI Col yields a constant value within the strain range of 0.1–20%. When the strain amount exceeds 10–20%, G′ suddenly drops, and the relative value of G′′ is increased. It can be judged that the viscous behavior became more prominent. At the concentration of 2 mg/mL or higher, the strain amount at G′ = G′′ (intersection of G′ and G”) shifted to a higher distortion side as the concentration increased. This indicates that the elastic behavior becomes more prominent as the concentration increases. At 1 mg/mL, no intersection point between G′ and G′′ was observed in the measured strain range. Therefore, the material was considered to be in a relatively viscous state.

However, L-ECM yielded a constant value in the strain range of 0.1 to 10% and exhibited a linear behavior in a narrower range compared to PI Col. In regard to the strain dispersion, the reduction of G′ and G′′ owing to the increase in the strain is considered to be nonlinear in terms of the material’s physical properties simply owing to its complicated internal structure. From Figure 5A,B, it can be observed that the strains exhibit nonlinearity (distortion values at which G′ and G′′ begin to decrease) decreased when the content was low. In this instance, the internal structure remarkably changed the rheological behavior. In other words, it is considered that the material that exhibits a more prominent nonlinearity has G′ > G′′, and the difference between them is larger. As the content increases, both G′ and G′′ increase, but the difference between them decreases. It is thought that this is due to an increase in the number of molecules, rendering the material difficult to deform owing to the secondary intermolecular bonds (such as hydrogen bonds), and as a result of the increased contribution of plastic deformation. Therefore, the constant elastic modulus region in the strain dispersion test justifies the dispersion stability of the substance in the solvent. It was suggested that the PI Col was excellent in terms of its dispersion stability since its stable strain range response spanned approximately 0.1–20% compared to approximate range of 0.1–10% for L-ECM. This indicates that solutes (such as collagen molecules) are difficult to aggregate and dissociate in the solvent. It is considered that the dispersion stability of L-ECM is lower than that of PI Col owing to the fact that L-ECM is not a completely homogeneous liquid but rather represents a state in which small particles are present. This is manifested by the fact that L-ECM is not clear or transparent, even after solubilization, but has a white and cloudy appearance (Figure 3C).

Similar to the PI Col, concentration increases led to increases in values of G′ and G′′, while the amount of strain indicating the intersection of each term shifted at higher strains. Subsequently, a comparison of L-ECM and PI Col at the same concentration showed that PI Col exhibited a more elastic behavior since the value of G′ of PI Col was higher than that of L-ECM. That is, at the same concentration, it can be said that the intermolecular friction of L-ECM is smaller than that of PI Col.

#### 2.3.2. Frequency Dispersion Test

In the frequency dispersion test, the vibration amplitude was constant and the vibration frequency was varied and measured. Since both samples showed linear behaviors in the vicinity of strains of approximately 5%, in the strain dispersion test, measurements in the frequency dispersion test were carried out at a strain level of 5% (Figure 5C,D).

In the case of the PI Col, increases in the G′ and G′′ values at increasing frequencies were confirmed. The rate of this increase was larger as the concentration decreased. This is thought to be due to an increase in the force applied to the polymeric chain. The increase in the G′ and G′′ values due to this frequency increase show an effect that is similar to the increase in the intermolecular interaction owing to concentration increases. Therefore, at low frequencies, it is considered that the viscoelastic behavior with relatively low intermolecular interactions, corresponding to low concentrations, affects the value of G′ and G′′.

Herein, the frequencies in the case where G′′ > G′ for concentrations of 1, 2, and 3 mg/mL of the PI Col were in the frequency regions of approximately 100, 10, and 1 Hz, respectively. This can also be explained by the fact that a dominant viscous behavior was elicited at low concentrations and low frequency conditions. That is, in the high frequency range, only short relaxation times affect G′′. Correspondingly, G′′ decreased and G′ increased. That is, the behavior resembled that of a liquid in the low frequency region and that of a solid in the high frequency region. It was suggested that the shifting of the point of intersection of G′ and G′′ to the high frequency side was due to the shortening of the typical relaxation time of the material.

Conversely, the increase in G′ and G′′ at higher frequencies was also confirmed in the L-ECM. However, G′ and G′′ did not intersect in the measured frequency range. Comparing the PI Col and the L-ECM at the same concentration, no noticeable differences were found between G′ and G′′ with respect to the frequency changes. Both PI Col and L-ECM showed that G′ > G′′ within the measured frequency ranges, thereby suggesting that this referred to a substrate that had a predominantly elastic behavior. However, the elasticity of PI Col was greater than that of L-ECM since there was a stronger intermolecular interaction in the PI Col compared to L-ECM at the same concentration, as also shown in the strain dispersion evaluation.

In alternative terms, the values of G′ and G′′ of PI Col were relatively close to each other, and the G′ value of L-ECM yielded a larger value than G′, because L-ECM had a higher elasticity compared to that of PI Col. Given the magnitude relationship between the absolute values of G′ and G′′, it seems that the entanglement of molecules on the L-ECM have a larger influence on the flow behavior than that for the PI Col. From the above frequency dispersion, the values of G′ and G′′ are relatively close to each other in the case of the PI Col, and the behavior of the L-ECM is relatively rubbery. That is, in L-ECM, molecular bonds are formed and the structure is expected to change or be extremely intertwined. In addition, it was suggested that the typical relaxation time of L-ECM is short since the decrease of G′′ at increasing frequencies was not confirmed in the L-ECM.

#### 2.3.3. Temperature Dependence

The temperature dependence (Figure 5E,F) was investigated at a strain of 5% and a frequency of 1 Hz. As a result of the measurement of the G′ and G′′ values in the course of the transition from low to high temperatures, PI Col elicited constant G′ and G′′ values as a function of temperature, but began to decrease after a certain temperature. This decline began at the porcine collagen denaturation temperature of 37 °C. It seems that the physical properties of the material changed due to protein denaturation. However, G′ and G′′ decreased gradually with temperature increases in the case of L-ECM, and a sudden decrease in the elastic modulus was observed at the same temperature as that observed in the case of the PI Col.

Based on the dynamic viscoelasticity evaluation of the L-ECM solution, it was confirmed that its elastic behavior became prominent and dominant in a concentration-dependent manner. In addition, this elicited behavior can be primarily attributed to the small number of molecular associations, and secondarily attributed to the weak intermolecular forces of the L-ECM constituent components. In the ImageJ analyses of the SDS-PAGE results, the proportion of α chains was larger in PI Col than in I Col, and the proportion of α chains was larger in L-ECM than in PI Col. The removal of the telopeptide decreased the fibrillogenic ability of collagen [20]. Based on this finding, it is considered that in the L-ECM there are few molecules associated with collagen chains, and the entanglement between molecules is poor. Secondly, it is a multicomponent mixed system. It has been reported that molecules, such as proteoglycans and fibronectin, affect the fibril formation of collagen due to their coexistence with collagen [21,22,23]. Thus, it is suggested that repulsion between molecules exists owing to the multicomponent system.

### 2.4. SEM Gel Observations

**Figure 6:**
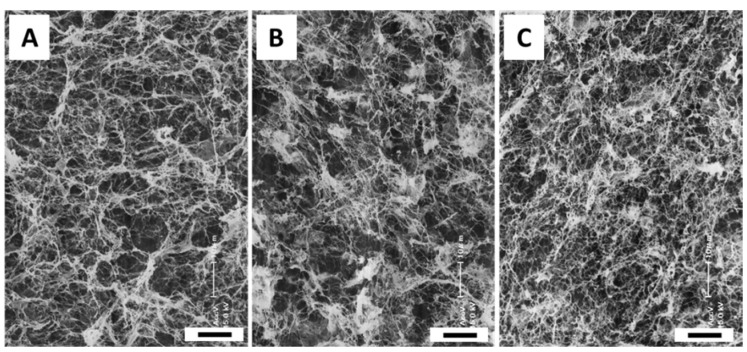
(**A**) SEM images of 3 mg/mL I Col gel, (**B**) 10 mg/mL L-ECM gel, and (**C**) 20 mg/mL L-ECM gel samples (all images are shown at a magnification of 1000 times. Scale bar = 10 µm).

SEM images of L-ECM and the I Col gel are shown in Figure 6. Since these photos were obtained by dehydration treatment, there is no guarantee that they accurately indicate the state of the ECM in the actual hydrogel. However, these images are important data to infer the skeleton structure of ECM gel [15,24]. Fibrous skeleton was observed in all samples. When comparing (A) the 3 mg/mL I Col gel and (B) the 10 mg/mL L-ECM gel samples, the densities of the fibers constituting the gels are comparable, whereas the fiber diameter in the 3 mg/mL I Col gel sample was thicker than that in the 10 mg/mL L-ECM gel sample. In addition, when comparing (B) the 10 mg/mL L-ECM and (C) the 20 mg/mL L-ECM gel samples, the fiber diameter was comparable. However, the fiber density was higher in the 20 mg/mL L-ECM gel sample compared to the 10 mg/mL L-ECM gel sample. Therefore, the diameters of the fibers constituting the gel of the 10 mg/mL L-ECM gel sample were smaller than those of the 3 mg/mL I Col gel sample, but the fiber densities were almost equal. In addition, the fiber density of the 20 mg/mL L-ECM gel was the highest at the conditions at which the tests were conducted. In other words, it was quantitatively found that the skeleton fiber diameter depends on the constituent components, and the fiber density depends on the concentration. In addition, these differences may be the result of other ECM components affecting the spontaneous self-assembly of type I collagen in L-ECM.

Spheroid, a spherical aggregate formed by the assembly of a large number of hepatocytes, expresses relatively better liver-specific functions [25,26]. However, hepatocytes embedded in spontaneously self-assembled collagen gel express liver functions that are equivalent to those of spheroids, even in the dispersed single cell state [9]. Furthermore, collagen gel-embedded hepatocyte spheroids express liver functions that are much higher than the above [9]. In other words, the in vivo-like self-assembled collagen fiber network is meaningful for cell culture and related tissue engineering applications. Therefore, L-ECM [11] gel, which has a synergistic effect with this collagen fiber network and other ECM components (such as GAGs capable of growth factor immobilization), is expected to be an important substrate promoting functional liver tissue formation.

### 2.5. Immunostaining of L-ECM Gel

**Figure 7:**
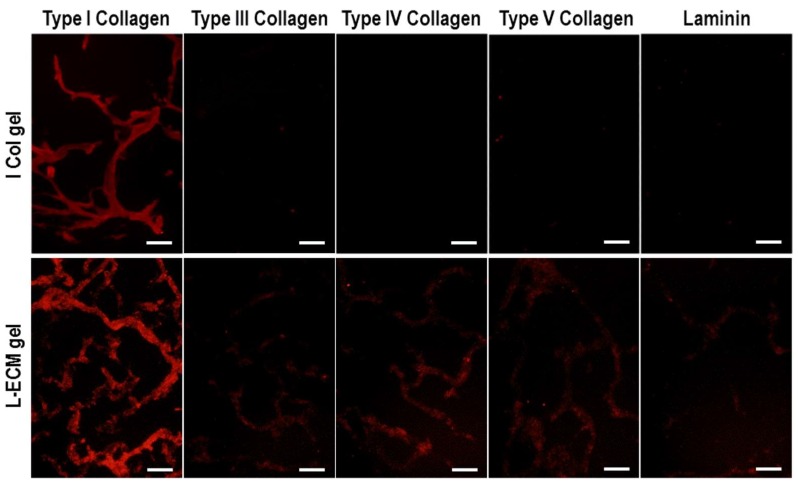
Fluorescent immunostaining images of I Col and L-ECM gel (scale bar = 50 µm).

Immunostaining images of L-ECM gel are shown in Figure 7. Fluorescence was confirmed only in the case where the type I collagen antibody was used in the I Col gel sample. Even if differences in fluorescence intensity were observed, red fluorescence was observed in the L-ECM gel when any antibody was used. Among all of the stained images, the strongest intensity in red fluorescence was detected when the type I collagen antibody was used. In other words, it was suggested that the L-ECM gel is a functional gel substratum that consisted of the ECM components that were originally present in the liver.

### 2.6. Rheological Properties of Gelation Behavior of L-ECM

**Figure 8:**
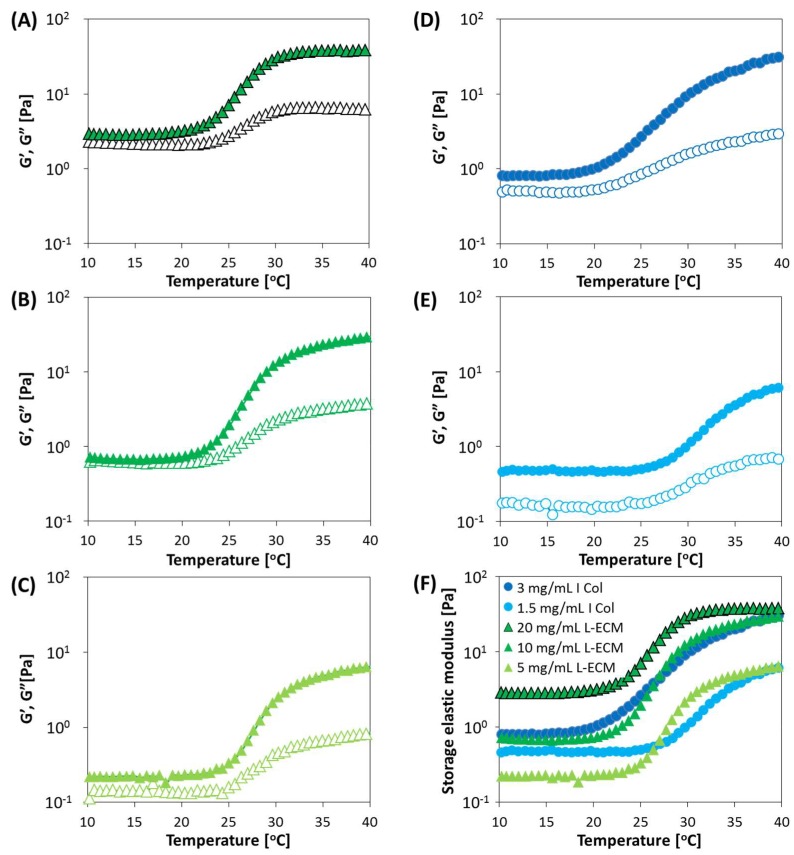
Change in G′ and G′′ at different concentrations of I Col (3 and 1.5 mg/mL) and L-ECM solutions (20, 10, and 5 mg/mL) with the rheometer measurements conducted in a DTR mode. (**A**) 20 mg/mL L-ECM, (**B**) 10 mg/mL L-ECM, (**C**) 5 mg/mL L-ECM, (**D**) 3 mg/mL I Col, (**E**) 1.5 mg/mL I Col, and (**F**) storage modulus for all tested conditions. (**A**–**E**) Open and closed symbols indicate storage and loss moduli, respectively (conditions of DTR: frequency, 1 Hz, and strain, 5%. Temperature was increased at 2 °C /min from 10 to 40 °C. The pH of all samples was adjusted to its value in physiological conditions).

The gelation behaviors of L-ECM and I Col are shown in Figure 8. For all of the studied conditions, an increase in the elastic modulus was confirmed as the temperature increased. In addition, the loss elastic modulus never exceeded the storage modulus within the measured temperature range. Here, similar gelation behaviors were observed between 10 mg/mL L-ECM and 3 mg/mL I Col solutions. In fact, in the preliminary study using rat L-ECM, the content of type I collagen was only about half of L-ECM (data not shown). Though there may be some effects of differences in species, their similarity in gelation kinetics can be deduced to be reasonable.

Normally, in the solution state, the loss elastic modulus yields a value higher than the storage modulus. Conversely, in the gel, the storage modulus exceeds the loss elastic modulus. This is due to the change to a solid state by gelation, whereby externally applied energy is stored and converted to a repulsive force. However, the earliest gel formation for L-ECM started at a concentration of 20 mg/mL. This is thought to be due to the frequent entanglement of components owing to an increase in the concentration and relatively early initiation of nucleation of fibers that occur at the initial stage of fibril formation.

### 2.7. Degradation Behavior of L-ECM Gel

**Figure 9:**
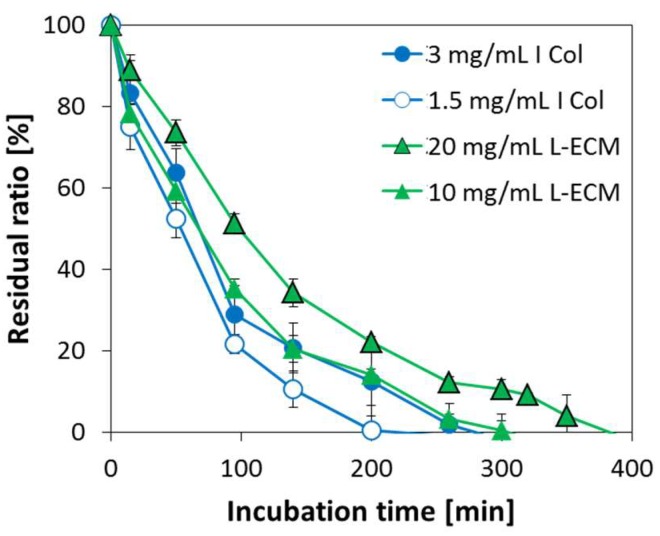
Degradation profiles of I Col gels (3 and 1.5 mg/mL) and L-ECM gels (20 and 10 mg/mL) using collagenase solution at a concentration of 0.05 mg/mL as a function of incubation time (*n* = 3, bars represent standard deviation).

The decomposition behaviors of L-ECM and I Col gel by collagenase are shown in Figure 9. Samples underwent digestion with collagenase at all tested conditions, and a decrease in gel weight over time was confirmed. In addition, solutions of 10 mg/mL L-ECM and 3 mg/mL I Col gel showed comparable degradation rates. Since L-ECM is composed of atelocollagen from which telopeptide had been removed, it can be easily degraded by proteases. The condition with the highest degradation rate was that for I Col gel with a concentration of 1.5 mg/mL, and the condition with the slowest degradation rate was that for L-ECM gel with a concentration of 20 mg/mL.

### 2.8. Stress on Compression of L-ECM Gel

**Figure 10:**
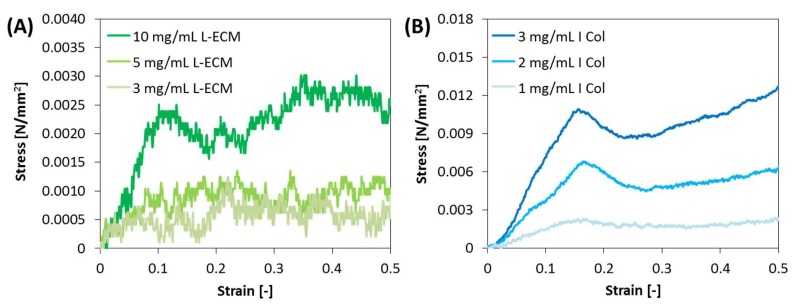
Average stress-strain responses (*n* = 3) of (**A**) L-ECM gel, and (**B**) I Col gel.

The stress-strain curves of L-ECM and I Col gel are shown in Figure 10, and the upper yield point and elastic modulus of each sample are shown in Table 1. The compression stress was detected at all tested conditions. In addition, a concentration-dependent compression response was obtained with I Col and L-ECM. Comparison of the upper yield points of I Col and L-ECM at each concentration yielded a maximum at 3 mg/mL for I Col, and its value was 0.0112 N/mm^2^. The elastic modulus was also the highest at the same conditions and its value was 0.0825 N/mm^2^. Comparing the I Col and L-ECM gels at the same concentration (3 mg/mL), the upper yield point and elastic modulus of I Col were 0.0112 and 0.0825 N/mm^2^, respectively, while those of L-ECM were 0.0008 and 0.0057 N/mm^2^, respectively. Furthermore, the gel compression test showed that the L-ECM gel with a concentration of 10 mg/mL elicited an inferior mechanical strength response compared to 3 mg/mL I Col.

The fiber diameters constituting the L-ECM gel were smaller than those for the I Col gel, as confirmed by SEM imaging. The degradation and gelling behaviors of the L-ECM gel were equivalent to those of I Col with a concentration of 3 mg/mL, but the mechanical strength of the gel was low. The reason for this is considered to be the fineness of the fiber diameters constituting the gel. In the rheological evaluation, the gelation behavior of the L-ECM gel sample with a concentration of 10 mg/mL was equivalent to that of the I Col gel sample with a concentration of 3 mg/mL. In general terms, the information obtained regarding the gelling behavior does not indicate the strength of the gel [27]. Therefore, through the comparison of the rheological evaluation results that showed the gelation behavior, a well as the compression test results that showed the mechanical strength, the L-ECM was found to elicit a gelation behavior similar to that of I Col, but the strength of the L-ECM gel was lower than that of the I Col gel.

However, it is possible to increase the mechanical strength. For example, this can be achieved using the EDC/NHS reaction in which ethyl (dimethylaminopropyl) carbodiimide (EDC) and N-hydroxysuccinimide (NHS) are used in combination [28]. In addition, collagen gel with high biocompatibility and excellent mechanical strength can be obtained by crosslinking the gel by transglutaminase [29,30]. It is also possible to manipulate the fiber density of the gel skeleton by increasing the concentration of the solution.

## 3. Conclusions

L-ECM was prepared as a liver-specific matrix substrate from the decellularized porcine liver. It had the characteristics of a pepsin-digested substrate and retained the various components contained in the native liver. L-ECM had a lower elasticity compared to pepsin-digested type I collagen, and showed an abrupt decrease in its elastic modulus at 37 °C. The elastic modulus increased with increasing temperatures (up to 40 °C), and the loss elastic modulus never exceeded the storage elastic modulus in the L-ECM gel. In addition, as the L-ECM gel concentration increased, decreases in the biodegradation rate and increases in the mechanical strength were confirmed. All of the elicited properties for L-ECM at a concentration of in 10 mg/mL were equivalent to collagen gel at a concentration of 3 mg/mL, and this concentration of collagen is commonly used for regenerative medicine and gel cultures.

It is expected that the obtained results will greatly contribute to the optimization of the scaffold for hepatic tissue engineering. Furthermore, the developed L-ECM is expected to be used as a substrate for functional hepatocyte culture. Lastly, the results will provide important information for understanding the liver-specific phenotypic expression of hepatocytes and formed liver tissue.

## 4. Materials and Methods

### 4.1. Decellularization of Porcine Liver

A healthy porcine liver (Kyudo, Saga, Japan) was depleted of blood in a fresh state with calcium and magnesium-free phosphate-buffered saline (CMF–PBS) and was cryopreserved at −80 °C until use. The porcine liver (Kyudo, Tosu, Japan) was sectioned in slices with a thickness of 2 mm and was decellularized by stirring at 4 °C with 1% Triton-X 100 (polyoxyethylene-p-isooctylphenol) (Sigma-Aldrich, St. Louis, MO, USA) in CMF-PBS. Thereafter, the decellularized liver was washed with CMF-PBS and further dialyzed against water. The dialysis membrane used was Spectra/Por 6 (MWCO: 1000, Spectrum Laboratories, Inc., Rancho Dominguez, CA, USA). After washing, lyophilization treatment was applied to obtain dried porcine liver ECM. The animal experimental protocol was reviewed and approved by the Ethics Committee on Animal Experiments of Kyushu University (A25-282-0, 21 Feb 2014).

### 4.2. Preparation of Liver-Specific ECM-Solubilized Substrate and Preparation of Hydrogel

Approximately 10 mg of lyophilized decellularized liver was placed in 1 mL of pepsin (Sigma-Aldrich) solution (1 mg/mL in 0.1 N HCl). Solubilized ECM derived from decellularized liver was obtained by the treatment with pepsin solution at 4 °C for 72 h. The pH of the ECM was adjusted to pH 3.0 by dialysis with Spectra/Por 6 (MWCO: 1000, Spectrum Laboratories, Inc.), and solubilized liver-specific ECM was obtained (L-ECM). DNA and glycosaminoglycan (GAG) contents were quantitatively analyzed using a Fluorescent DNA Quantification Kit (Bio-Rad Laboratories Inc., Hercules, CA, USA) and GAGs quantitative kit (Euro Diagnostica AB, Malmö, Sweden) assay according to the recommended protocol. L-ECM was mixed with concentrated minimum essential medium eagle (MEM) (×10) and buffer (47.7 mg HEPES/mL, 0.08N NaOH) at a volume ratio of 8:1:1 (*v*/*v*) and was kept on ice. The solution formed a gel after incubation at 37 °C for 30 min by assembling itself into a three-dimensional network.

### 4.3. Immunostaining

Sections with a thickness of 10 μm were prepared using a freezing microtome (CM 1100: Leica Microsystems GmbH, Wetzlar, Germany) and immobilized by immersion in 4% formaldehyde for 10 min. It was then washed with CMF-PBS and was immersed in CMF-PBS that was supplemented with 1% bovine serum albumin (BSA) (Wako, Osaka, Japan) for 30 min for blocking treatment. Primary antibody diluted with CMF-PBS was supplemented with 1% BSA was then added in a dropwise manner to the sample. It was left overnight at 4°C. After washing with CMF-PBS supplemented with 1% BSA, a secondary antibody was diluted with CMF-PBS that was supplemented with 1% BSA. It was added drop-by-drop to the sample and was allowed to stand at room temperature for 1 h. All operations after the addition of the secondary antibody were conducted using light shielding. Subsequently, the sample was washed with CMF-PBS supplemented with 1% BSA, covered with a cover glass, and observed with a fluorescence microscope. Details of the antibodies used were as follows. All primary antibodies were rabbit-derived antibodies against rat proteins. Anti-collagen type I was purchased from Rockland Antibodies and Assays (Limerick, PA, USA). Anti-collagen type III, anti-collagen type IV, and anti-collagen type V were purchased from Abbiotech (San Diego, CA, USA). Anti-laminin was purchased from Bioss Antibodies (Woburn, MA, USA). In addition, goat anti-rabbit IgG TRITC-conjugated antibody (ThermoFisher Scientific-Invitrogen, Waltham, MA, USA) was used as a secondary antibody.

### 4.4. Molecular Weight Distribution of L-ECM

SDS-PAGE was used for the investigation of molecular weight distributions. Electrophoresis samples of L-ECM at a concentration of 0.75 mg/mL, acid-solubilized porcine type I collagen (I Col), and pepsin-solubilized porcine type I collagen (PI Col) (Nitta Gelatin Inc.) were prepared. A 5% acrylamide gel was prepared and each sample was electrophoresed at 200 V with 40 mA. Furthermore, by analyzing the SDS-PAGE image obtained by ImageJ, the proportion of each molecular weight in all molecules was calculated.

### 4.5. SEM Gel Observations

Samples of L-ECM at concentrations of 10 and 20 mg/mL as well as I Col samples at concentrations of 3 mg/mL-type were adjusted to neutral pH by mixing with reconstitution buffer (47.7 mg HEPES/mL, 0.08 N NaOH) and ×10 MEM at a ratio of 8:1:1. Each adjusted sample had a concentration that was equal to four-fifths of the above concentration. The solution formed a gel after incubation at 37 °C for 30 min by assembling itself into a three-dimensional network. The sample was substituted for ethanol and t-butanol (Wako) for dehydration. After replacement, the liver was allowed to stand at 4 °C, and was then dried using a vacuum pump. The morphological structure of the obtained dried sample was observed with a scanning electron microscope (SEM, SS 550: Shimadzu Co., Kyoto, Japan).

### 4.6. Rheology of L-ECM

Pepsin-solubilized I col (PI Col) samples at concentrations of 1, 2, and 3 mg/mL were used as the relative evaluation conditions for L-ECM samples at concentrations of 3, 5, and 10 mg/mL. Additionally, the apparatus used was a viscoelasticity measuring apparatus MCR (Anton Paar, Graz, Austria). In the analysis, the storage modulus (G′, elastic term) and loss modulus (G′′, viscous term) were determined and expressed as respective graphs with respect to distortion, frequency, and temperature. (1) Distortion dispersion (dynamic strain sweep method, DSS) was measured with a cone plate (0.5°, diameter 50 mm), and the strain was changed from 0.1 to 1000% with a frequency of 1 Hz and a temperature of 10 °C for these measurements. (2) Frequency dispersion (dynamic frequency sweep method, DFS) was measured using a cone plate (0.5°, diameter 50 mm) by changing the frequency from 1 to 100 Hz with a strain of 5% and a temperature of 10 °C. (3) Temperature dispersion (dynamic temperature ramping method, DTR) was obtained by increasing the temperature from 10 to 50 °C at 1 °C/min using a parallel plate (50 mm in diameter) at a frequency of 1 Hz and a strain of 5%.

In the preliminary study, L-ECM prepared using powders stored at room temperature for 1 week gelled promptly under neutral conditions. However, when powders stored at 37 °C were used, the gelling ability of L-ECM remarkably decreased (data not shown). In other words, L-ECM before gelation was affected by insufficient stability at body temperature. Therefore, in (1) and (2), measurement was carried out at 10 °C.

### 4.7. Rheology of Gelation Behavior of L-ECM

In order to investigate the gelation behavior of L-ECM, rheological evaluation was performed. Samples of L-ECM at the concentrations of 5, 10, and 20 mg/mL, as well as I Col samples at the concentrations of 1.5 and 3 mg/mL, were adjusted to neutral pH by mixing with reconstitution buffer and ×10 MEM at a ratio of 8:1:1. The storage elastic moduli of these samples were measured by linearly increasing the temperature from 10 to 40 °C at 2 °C/min using a rheometer at a frequency of 1 Hz. A parallel plate (diameter: 50 mm) was used for the measurement.

### 4.8. Degradation Behavior of L-ECM Gel

Collagenase digestion was performed to investigate the degradation properties of the gel. Samples of L-ECM at the concentrations of 10, 20 mg/mL, or I Col samples at the concentrations of 1.5, 3 mg/mL, were adjusted to neutral pH by mixing them with reconstitution buffer and ×10 MEM at a ratio of 8:1:1. Each adjusted sample had a concentration that was equal to four-fifths of the above concentration. A gel volume of 500 μL was shaken in 10 mL of 0.5 mg/mL collagenase (Wako)/0.05 mg/mL trypsin inhibitor (Wako) solution mixture, and the residual weight of the gel was measured as a function of time. The difference from the initial weight was calculated and evaluated as the manifestation of its degradation characteristics.

### 4.9. Stress on the Compression of L-ECM Gel

The mechanical properties of the gel against compression were evaluated. The mechanical strength of the gel was evaluated by determining the stress-strain curve, and by investigating the stress at the upper yield point obtained by increasing the load beyond the elastic limit, thereby identifying the plastic deformation point of the gel. In addition, the elastic modulus was obtained from the linearly increasing parts of the stress-strain curves. Samples of L-ECM solutions with concentrations of 3, 5, and 10 mg/mL, or I Col samples with concentrations of 1, 2, and 3 mg/mL, were added to a 96-well plate at volumes of 300 μL/well and were incubated at 37 °C overnight. The stress on the compression of the gel was measured using a load measuring machine (LTS-50N-S100: Minebea Co., Nagano, Japan). The height of the gel was measured and a stress-strain curve was generated.

## Figures and Tables

**Table 1:** Upper yield point and elastic modulus of I Col and L-ECM gels.

Sample	Concentration (mg/mL)	Upper Yield Point (N/mm^2^)	Elastic Modulus (N/mm^2^)
I Col	3	0.0112	0.0825
	2	0.0069	0.0428
	1	0.0025	0.0175
L-ECM	10	0.0026	0.0318
	5	0.0014	0.0153
	3	0.0008	0.0057

## References

[1] Sasaki S., Funamoto S., Hashimoto Y., Kimura T., Honda T., Hattori S., Kobayashi H., Kishida A., Mochizuki M. (2009). In vivo evaluation of a novel scaffold for artificial corneas prepared by using ultrahigh hydrostatic pressure to decellularize porcine corneas. Mol. Vis..

[2] Poh M., Boyer M., Solan A., Dahl S.L., Pedrotty D., Banik S.S., McKee J.A., Klinger R.Y., Counter C.M., Niklason L.E. (2005). Blood vessels engineered from human cells. Lancet.

[3] Uygun B.E., Soto-Gutierrez A., Yagi H., Izamis M.L., Guzzardi M.A., Shulman C., Milwid J., Kobayashi N., Tilles A., Berthiaume F. (2010). Organ reengineering through development of a transplantable recellularized liver graft using decellularized liver matrix. Nat. Med..

[4] Baptista P.M., Siddiqui M.M., Lozier G., Rodriguez S.R., Atala A., Soker S. (2011). The use of whole organ decellularization for the generation of a vascularized liver organoid. Hepatology.

[5] Butter A., Aliyev K., Hillebrandt K.H., Raschzok N., Kluge M., Seiffert N., Tang P., Napierala H., Muhamma A.I., Reutzel-Selke A. (2018). Evolution of graft morphology and function after recellularization of decellularized rat livers. J. Tissue Eng. Regen. Med..

[6] Kojima H., Yasuchika K., Fukumitsu K., Ishii T., Ogiso S., Miyauchi Y., Yamaoka R., Kawai T., Katayama H., Yoshitoshi-Uebayashi E.Y. (2018). Establishment of practical recellularized liver graft for blood perfusion using primary rat hepatocytes and liver sinusoidal endothelial cells. Am. J. Transplant..

[7] Shirakigawa N., Takei T., Ijima H. (2013). Base structure consisting of an endothelialized vascular-tree network and hepatocytes for whole liver engineering. J. Biosci. Bioeng..

[8] Pan J., Yan S., Gao J.J., Wang Y.Y., Lu Z.J., Cui C.W., Zhang Y.H., Wang Y., Meng X.Q., Zhou L. (2016). In-vivo organ engineering: Perfusion of hepatocytes in a single liver lobe scaffold of living rats. Int. J. Biochem. Cell Biol..

[9] Ijima H. (2010). Practical and functional culture technologies for primary hepatocytes. Biochem. Eng. J..

[10] Shirakigawa N., Ijima H. (2013). Nucleus number in clusters of transplanted fetal liver cells increases by partial hepatectomy of recipient rats. J. Biosci. Bioeng..

[11] Nakamura S., Ijima H. (2013). Solubilized matrix derived from decellularized liver as a growth factor-immobilizable scaffold for hepatocyte culture. J. Biosci. Bioeng..

[12] Loneker A.E., Faulk D.M., Hussey G.S., D’Amore A., Badylak S.F. (2016). Solubilized liver extracellular matrix maintains primary rat hepatocyte phenotype in-vitro. J. Biomed. Mater. Res. A.

[13] Saheli M., Sepantafar M., Pournasr B., Farzaneh Z., Vosough M., Piryaei A., Baharvand H. (2018). Three-Dimensional Liver-derived Extracellular Matrix Hydrogel Promotes Liver Organoids Function. J. Cell Biochem..

[14] Damania A., Kumar A., Teotia A.K., Kimura H., Kamihira M., Ijima H., Sarin S.K., Kumar A. (2018). Decellularized liver matrix-modified cryogel scaffolds as potential hepatocyte carriers in bioartificial liver support systems and implantable liver constructs. ACS Appl. Mater. Interfaces.

[15] Lee J.S., Shin J., Park H.M., Kim Y.G., Kim B.G., Oh J.W., Cho S.W. (2014). Liver extracellular matrix providing dual functions of two-dimensional substrate coating and three-dimensional injectable hydrogel platform for liver tissue engineering. Biomacromolecules.

[16] Zhang Y., He Y., Bharadwaj S., Hammam N., Carnagey K., Myers R., Atala A., Dyke M.V. (2009). Tissue-specific extracellular matrix coatings for the promotion of cell proliferation and maintenance of cell phenotype. Biomaterials.

[17] DeQuach J.A., Lin J.E., Cam C., Hu D., Salvatore M.A., Sheikh F., Christman K.L. (2012). Injectable skeletal muscle matrix hydrogel promotes neovascularization and muscle cell infiltration in a hindlimb ischemia model. Eur. Cells Mater..

[18] You J., Park S.A., Shin D.S., Patel D., Raghunathan V.K., Kim M., Murphy C.J., Tae G., Revzin A. (2013). Characterizing the effects of heparin gel stiffness on function of primary hepatocytes. Tissue Eng. Part A.

[19] Bairati A., Garrone R. (1985). Biology of Invertebrate and Lower Vertebrate Collagen.

[20] Yoshimura K., Terashima M., Hozan D., Shirai K. (2000). Preparation and Dynamic Viscoelasticity Characterization of Alkali-Solubilized Collagen from Shark Skin. J. Agric. Food Chem..

[21] Payne A.R., Whittaker R.E. (1971). Low strain dynamic properties of filled rubbers. Rubber Chem. Technol..

[22] Freakly P.K., Payne A.R. (1978). Theory and Practice of Engineering with Rubber.

[23] Snowdend J.M., Swann D.A. (1979). The formation and thermal stability of in vitro assembled fibrils from acid-soluble and pepsin-treated collagens. Biochem. Biophys. Acta.

[24] Lang R., Stern M.M., Smith L., Liu Y., Bharadwaj S., Liu G., Baptista P.M., Bergman C.R., Soker S., Yoo J.J. (2011). Three-dimensional culture of hepatocytes on porcine liver tissue-derived extracellular matrix. Biomaterials.

[25] Ijima H., Matsushita T., Nakazawa K., Fujii Y., Funatsu K. (1998). Hepatocyte spheroids in polyurethane foams: Functional analysis and application for a hybrid artificial liver. Tissue Eng..

[26] Ijima H., Nakazawa K., Mizumoto H., Matsushita T., Funatsu K. (1998). Formation of a spherical multicellular aggregate (spheroid) of animal cells in the pores of polyurethane foam as a cell culture substratum and its application to a hybrid artificial liver. J. Biomater. Sci. Polym. Ed..

[27] Yoshimura K., Chonan Y., Shirai K. (1997). Reactivity of Shark, Pig, and Bovine Skin Collagens with Formaldehyde and Basic Chromium Sulfate. Anim. Sci. Technol. (Jpn.).

[28] Liao J., Joyce E.M., Sacks M.S. (2008). Effects of decellularization on the mechanical and structural properties of the porcine aortic valve leaflet. Biomaterials.

[29] Piez K.A., Piaz K.A., Reddi A.H. (1988). Extracellular Matrix Biochemistry.

[30] Bond M.D., Van Wart H.E. (1984). Characterization of the individual collagenases from Clostridium histolyticum. Biochemistry.

